# The species distribution, antimicrobial resistance and risk factors for poor outcome of coagulase-negative staphylococci bacteraemia in China

**DOI:** 10.1186/s13756-019-0523-5

**Published:** 2019-04-24

**Authors:** Jiewei Cui, Zhixin Liang, Zhenfei Mo, Jianpeng Zhang

**Affiliations:** 1 Department of Respiratory Medicine, the First Medical Centre of Chinese PLA General Hospital, Fuxing Road No. 28, Beijing, 100853 China; 2 Department of Respiratory Medicine, the Third Medical Centre of Chinese PLA General Hospital, Yongding Road No.69, Beijing, 100853 China

**Keywords:** Coagulase-negative staphylococci, Bacteraemia, Species distribution, Antimicrobial resistance, Risk factors

## Abstract

**Objective:**

Coagulase-negative staphylococci (CoNS) are one of the major opportunistic pathogens and the incidence of CoNS bacteraemia is increasing. However, most of the CoNS-positive blood cultures are contaminants rather than true CoNS bacteraemia. In order to minimize contamination, we defined true CoNS bacteraemia as the patient that has two or more identical CoNS-positive blood cultures drawn within 48 h in this study and the objective of this study was to analyse the species distribution and antibiotic resistance and to identify risk factors for 30-day mortality of the true CoNS-bacteraemia.

**Method:**

By reviewing the electronic medical database, this study retrospectively analysed patients diagnosed as CoNS bacteraemia by blood cultures in a comprehensive tertiary care hospital in China from January 1, 2014, to December 31, 2017.

**Result:**

A total of 1241 patients with 1562 episodes of CoNS-positive blood cultures were recorded in the database but only 157 patients were finally diagnosed as true CoNS bacteraemia after contaminants were excluded. All these 157 patients (12.7%, 157/1241) had bacteraemia-related clinical symptoms. Among the 157 patients, the most common species were *Staphylococcus hominis* (40.8%), *Staphylococcus epidermidis* (36.3%) and *Staphylococcus capitis* (11.5%). The antimicrobial susceptibility tests showed that all CoNS had a high rate of resistance to penicillin (94.9%), oxacillin (93.6%) and erythromycin (92.4%). Resistance to gentamicin (22.3%) and rifampicin (10.8%) was low, and none of the bacteria were resistant to vancomycin or linezolid. The 30-day mortality of patients with CoNS bacteraemia was up to 12.7% (20/157), and the multivariate logistics regression analysis showed that chronic renal failure (OR 5.9, 95% CI 1.6–21.5, *p* = 0.007) and chronic liver failure (OR 4.0, 95% CI 1.2–13.1, *p* = 0.024) were both the significant independent risk factors for the 30-day mortality of CoNS bacteraemia.

**Conclusion:**

*Staphylococcus hominis* and *Staphylococcus epidermidis* were the most common species in CoNS bacteraemia. All CoNS had high multi-drug resistance, but gentamicin and rifampicin had a relatively lower resistance and could be considered as alternative antibiotics for anti-CoNS bacteraemia in addition to vancomycin and linezolid. Additionally, patients with chronic renal failure or chronic liver failure have a higher 30-day mortality after the onset of CoNS bacteraemia.

## Background

Coagulase-negative staphylococci (CoNS) are the most abundant common microbial species of the human skin and mucous membranes, and they are one of the major health care-associated opportunistic pathogens [1, 2]. CoNS can easily attach and grow on the surface of medical devices through a slime layer that has a muco-polysaccharide structure [3–5]. CoNS may cause bacteraemia when medical devices penetrate the skin and the mucous membrane barrier and enter the blood [6]. With the increasing use of invasive medical devices, such as the central venous catheter (CVC) and periprosthetic joint [7, 8], the incidence of health care-associated CoNS bacteraemia is increasing [8–10].

CoNS are usually multi-drug resistant [11, 12]. The antimicrobial susceptibility of CoNS is critical for clinical empirical antibiotic therapy. Some recent studies suggested that CoNS were highly resistant to penicillin (PEN) (94.7%), oxacillin (OXA) (90.7%), and erythromycin (ERY) (85.3%), and more than 50% were resistant to cephalosporins, aminoglycosides and quinolones [13, 14]. However, most CoNS-positive blood cultures are false positives because CoNS could easily lead to blood culture contamination, and previous study suggested that the false-positive rates of CoNS-positive blood cultures were up to 90.9% if only one single CoNS-positive blood culture was present within 48 h [15]. According to false-positive blood cultures, antibiotics against CoNS may be overused, and patient care costs may be increased [16]. However, previous study had also pointed out that the false-positive rate dropped to 8.8% if an additional identical CoNS-positive blood culture was present within 48 h after the first CoNS-positive blood culture [15]. And up to now, there are rare studies on CoNS bacteraemia that had two or more identical CoNS-positive blood cultures drawn within 48 h, although there were many studies on CoNS bacteraemia.

With the aim to determine the species distribution, antibiotic resistance and to identify risk factors for poor outcome of true CoNS-bacteraemia that had two or more identical CoNS-positive blood cultures drawn within 48 h, we conducted the present study in a tertiary care hospital in China.

## Methods

### Hospital setting

This study was undertaken at the Chinese People’s Liberation Army General Hospital (PLAGH), which is a 2200-bed, tertiary care hospital in Beijing, China. This centre is a comprehensive hospital with medical, health care, teaching, and scientific research accreditation that serves all military and nonmilitary personnel from all regions in China.

### Study design

This was a retrospective study of hospitalized adult patients with CoNS-positive blood cultures in PLAGH from January 1, 2014, to December 31, 2017. All data were gathered by reviewing an ongoing prospective health care-associated infection surveillance electronic database of all positive blood cultures from all departments except paediatrics in the hospital.

### Case definitions

The criteria used to classify CoNS as a pathogen and rule out contaminants in blood culture refer to a laboratory-based algorithm [15]. The patients with CoNS bacteraemia involved in the study met the inclusion criteria and the exclusion criteria as follows. Inclusion criteria: (1) The patients were aged ≥18 years, patients under the age of 18 were excluded since risk factors and antimicrobial therapy for bloodstream infections among children are special. (2) The patients had at least two or more CoNS-positive blood cultures drawn within 48 h, and the species were identical; however, only the first episode was included. (3) The patient must have had clinical symptoms or markers of inflammation associated with bacteraemia, e.g., fever (> 38 °C), chills or elevated C-reactive protein (CRP). Exclusion criteria: (1) Patients who were pregnant or AIDS were excluded. (2) The onset of CoNS bacteraemia within the first 48 h after admission was excluded to ensure health care-associated infection [17]. (3) The patients with non-CoNS bacteraemia were excluded.

### Identification of isolates and antimicrobial susceptibility test

Blood cultures were performed at the hospital’s clinical microbiology laboratory. The blood culture was processed using the BacT/ALERT 3D Microbial Detection System (Becton-Dickinson, Sparks, MD, USA), and the species identification was performed using the VITEK 2 System (BioMérieux, Marcy 1′Étoile, France). Gram staining and coagulase testing were performed during the identification process. After the species identification, the antimicrobial susceptibility of isolated species was determined using standard procedures. Antibiotics for susceptibility: penicillin (PEN), oxacillin (OXA), erythromycin (ERY), cefoxitin (CEF), clindamycin (CLN), levofloxacin (LEV), moxifloxacin (MOX), trimethoprim/sulfamethoxazole (SUL), gentamicin (GEN), rifampicin (RIF), vancomycin (VAN) and linezolid (LIN). Methicillin resistance was determined by anti-OXA, which has a similar structure and antimicrobial susceptibility but better stability than PEN. The implementation standard of the antimicrobial susceptibility test was performed in accordance with the Clinical and Laboratory Standards Institute (CLSI) standards revised on January 1st, 2014 [18].

### Data collection

The demographic and clinical characteristics were collected as follows: (1) Demographics: gender, age, body mass index (BMI), the onset date of CoNS bacteraemia (the date when the first CoNS-positive blood culture was drawn), total hospital stay length prior to the onset of CoNS bacteraemia, acute physiology and chronic health evaluation (APACHE) II scores and quick sepsis related organ failure assessment (qSOFA) scores which were evaluated based on medical records within 24 h after the onset of CoNS bacteraemia. (2) Treatments within 7 days prior to the onset of CoNS bacteraemia: indwelling CVC, surgery (including trauma), invasive mechanical ventilation, chemotherapy, renal replacement therapy including haemofiltration and dialysis, total parenteral nutrition, indwelling urinary catheter, glucocorticoid therapy (methylprednisolone> 1 mg/kg/day), and broad-spectrum antibiotics (defined as fourth-generation cephalosporins and carbapenems). (3) Comorbidities at the onset of CoNS bacteraemia: cardiovascular disease, pneumonia, cerebrovascular disease, diabetes mellitus, chronic renal failure, chronic liver failure, solid tumour, haematologic malignancy, and neutropenia. (4) Laboratory test results: inflammatory markers within 24 h after the onset of CoNS bacteraemia included the number of leukocytes, the number of neutrophils, and CRP. In addition, alanine aminotransferase (ALT) and serum creatinine (SCr) levels for assessing liver and renal dysfunction were also collected. (5) Antibiotics used for empirical antibiotic therapy within 72 h after the onset of CoNS bacteraemia. (6) Outcomes: survival or non-survival on the 30th day after the onset of CoNS bacteraemia was used to calculate 30-day mortality (all the data about outcomes not in the database were obtained by telephone follow-up).

The microbiological data collected included the isolated species of CoNS bacteraemia and their antimicrobial susceptibility results. According to the antimicrobial susceptibility results, empirical antibiotic therapy within 72 h after the onset of CoNS bacteraemia was considered appropriate if the antibiotic was sensitive. Additionally, empirical antibiotic therapy was also considered appropriate if the clinical symptom (temperature > 38 °C) recovered to normal (temperature < 38 °C) within 48 h after empirical antibiotic therapy.

### Statistical analysis

Continuous variables were described as the mean (standard division SD) or median (interquartile range, IQR), as appropriate according to normality. Categorical variables were described as frequency counts and percentages (n, %). In the analysis of risk factors for 30-day mortality, univariate regression analysis was used to initially screen for possible risk factors. All variables significant in univariate analysis were included in multivariate logistic and Cox regression analysis to analyse independent risk factors, but all the variables included in multivariate regression analysis should been performed statistical analysis on multicollinearity and correlation between risk factors to exclude the composite variables. All statistical analysis were performed using SPSS 22.0 software (IBM Corp., Armonk, NY, USA) and all results with a 2-tailed *p*-value< 0.05 were considered significant.

## Results

### Details on patient enrolment

**Fig. 1:**
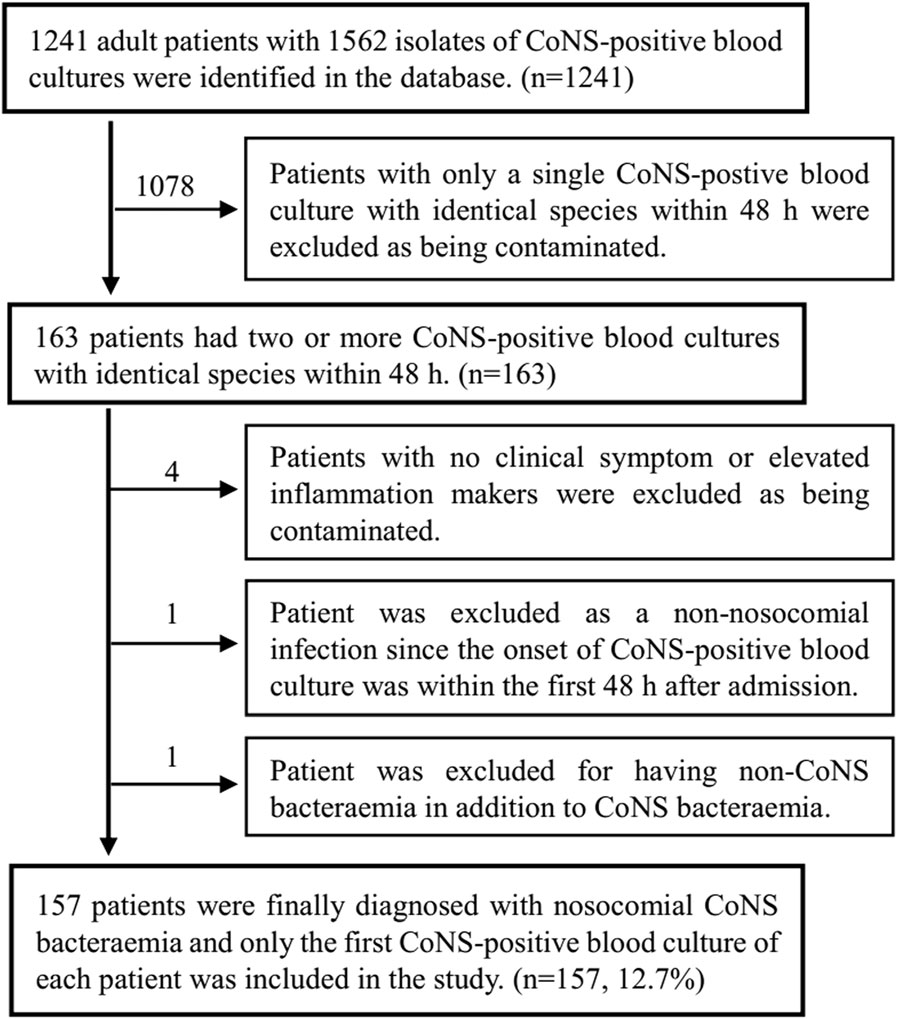
Details on patient enrolment in this study

A total of 1241 adult patients with 1562 isolates of CoNS-positive blood cultures were identified in the ongoing prospective health care-associated infection surveillance electronic database from 2014 to 2017. Among them, only 163 patients had two or more CoNS-positive blood cultures with identical species within 48 h. Four patients with no clinical symptoms or elevated inflammation markers were excluded, and one patient was excluded as a non-health care-associated infection since the onset of CoNS-positive blood culture was within the first 48 h after admission. In addition, one patient was excluded for having non-CoNS bacteraemia in addition to CoNS bacteraemia. Finally, 157 patients were diagnosed as health care-associated CoNS bacteraemia and were included in the study, which was 12.7% (157/1241) of all patients with CoNS-positive blood cultures. Among the 157 patients, the onset of the first CoNS bacteraemia was on January 9th, 2014. Details on patient enrolment were shown in Fig. 1.

### Microbiology and antimicrobial susceptibility

The distribution and antimicrobial susceptibility of the species isolated among the 157 patients with CoNS bacteraemia are shown in Table 1. Among the 157 patients, the most common species was *Staphylococcus hominis* (64 isolates, 40.8%), followed by *Staphylococcus epidermidis* (57 isolates, 36.3%), *Staphylococcus capitis* (18 isolates, 11.5%) and *Staphylococcus haemolyticus* (12 isolates, 7.6%).

**Table 1:** The distribution of species and their antimicrobial susceptibility in CoNS bacteraemia

Species	No of isolates (n, %)	Rate of resistance to a panel of 12 antibiotics (%)											
		PEN	OXA	ERY	CEF	CLN	LEV	MOX	SUL	GEN	RIF	VAN	LIN
Total	157 (100%)	94.9	93.6	92.4	78.3	73.2	72.6	71.3	59.9	22.3	10.8	0	0
*S. hominis*	64 (40.8%)	96.9	92.2	98.4	84.4	85.9	79.7	79.7	62.5	9.4	9.4	0	0
*S. epidermidis*	57 (36.3%)	96.5	96.5	87.7	75.4	61.4	61.4	59.6	71.9	28.1	15.7	0	0
*S. capitis*	18 (11.5%)	77.8	83.3	88.9	61.1	77.8	72.2	66.7	11.1	16.7	0	0	0
*S. haemolyticus*	12 (7.6%)	100	100	91.7	91.7	50	100	100	66.7	75	16.7	0	0
*S. saprophyticus* ^*a*^	3	3	3	3	1	2	0	0	1	0	0	0	0
*S. cohnii* ^*a*^	2	2	2	1	2	2	2	2	2	0	0	0	0
*S. sciuri* ^*a*^	1	1	1	1	1	1	1	1	0	1	0	0	0

*S*. Staphylococcus, *PEN* penicillin, *OXA* oxacillin, *ERY* erythromycin, *CEF* cefoxitin, *CLN* clindamycin, *LEV* levofloxacin, *MOX* moxifloxacin, *SUL* trimethoprim/sulfamethoxazole, *GEN* gentamicin, *RIF* rifampicin, *VAN* vancomycin and *LIN* linezolid

^a^The data were described as “n” since each frequency was too small

Overall, among all the species isolated, a high rate of resistance to OXA (93.6%), PEN (94.9%) and ERY (92.4%) was observed, but no species were resistant to LIN and VAN. The resistance to methicillin was determined by OXA. Thus, the proportion of resistance to methicillin ranged from 83.3% (*Staphylococcus capitis*) to 100% (*Staphylococcus haemolyticus*), and methicillin-resistant *Staphylococcus epidermidis* (MRSE) accounted for 96.5%. The antibiotic resistance details of each species are shown in Table 1.

### Demographic and clinical characteristics of the patients

The demographic and clinical characteristics of the 157 patients were shown in Table 2. The median age was 64 years (IQR, 48.0–80.0), age ≥ 60 years accounted for 58.6% (92/157), and 108 (68.8%) patients were male.

**Table 2:** Clinical Characteristics and univariate logistics analysis of risk factors for 30-day mortality of patients diagnosed with CoNS bacteraemia

	Total *n* = 157	Survival *n* = 137	Non-survival *n* = 20	OR	95% CI	*p*-value
Age	64.0 (48.0–80.0)	62.0 (48.0–80.0)	72.0 (62.8–80.2)	1.0	1.0–1.0	0.112
≥60 years	92 (58.6%)	76 (55.5%)	16 (80.0%)	3.2	1.0–10.1	0.046*
< 60 years	65 (41.4%)	61 (44.5%)	4 (20.0%)			
Gender						
female	49 (31.2%)	42 (30.7%)	7 (35%)	1.2	0.5–3.3	0.696
male	108 (68.8%)	95 (69.3%)	13 (65.0%)			
Residence in ICU	66 (42.0%)	52 (38.0%)	14 (70.0%)	3.8	1.4–10.5	0.010*
APACHE II score	12.0 (6.0–20.0)	11.0 (1.0–36.0)	20.0 (6.0–32.0)	1.1	1.0–1.2	0.002*
qSOFA score						
2 and 3	36 (22.9%)	26 (19.0%)	10 (50.0%)	4.3	1.6–11.3	0.004*
0 and 1	121 (77.1%)	111 (81.0%)	10 (50.0%)			
Prior hospital stay length	15.0 (4.0–35.0)	13.0 (4.0–31.0)	30.5 (5.8–46.2)	2.8	1.0–1.0	0.707
≥28 days	53 (33.8%)	42 (31%)	11 (55%)	2.8	1.1–7.2	0.036*
< 28 days	104 (66.2%)	95 (69.3%)	9 (45.0%)			
Comorbidities						
Cardiovascular disease	95 (60.5%)	82 (59.9%)	13 (65.0%)	1.2	0.5–3.3	0.661
Pneumonia	73 (46.5%)	59 (43.1%)	14 (70.0%)	3.1	1.1–8.5	0.030*
Cerebrovascular disease	46 (29.3%)	42 (30.7%)	4 (20.0%)	0.6	0.2–1.8	0.333
Diabetes mellitus	36 (22.9%)	32 (23.4%)	4 (20.0%)	0.8	0.3–2.6	0.739
Chronic liver failure	29 (18.5%)	20 (14.6%)	9 (45.0%)	4.8	1.8–13.0	0.002*
Chronic renal failure	27 (17.2%)	18 (13.1%)	9 (45.0%)	5.4	2.0–14.9	0.001*
Solid tumor	28 (17.8%)	24 (17.5%)	4 (20.0%)	1.2	0.4–3.8	0.787
Hematologic malignancy	11 (7.0%)	9 (6.6%)	2 (10.0%)	1.6	0.3–7.9	0.577
Neutropenia	12 (7.6%)	11 (8.0%)	1 (5.0%)	0.6	0.1–4.9	0.637
Prior treatments						
Indwelling CVC	88 (56.1%)	74 (54.0%)	14 (70.0%)	2.0	0.7–5.5	0.184
Indwelling urinary catheter	67 (42.7%)	57 (41.6%)	10 (50.0%)	1.4	0.5–3.6	0.480
Invasive MV	38 (24.2%)	33 (24.1%)	5 (25.0%)	1.1	0.4–3.1	0.929
Broad-spectrum antibiotics	31 (19.7%)	45 (32.8%)	6 (30.0%)	0.9	0.3–2.4	0.800
Surgery	28 (17.8%)	26 (19.0%)	2 (10.0%)	0.5	0.1–2.2	0.337
Renal replacement therapy	9 (5.7%)	7 (5.1%)	2 (10.0%)	2.1	0.4–10.7	0.389
Chemotherapy	4 (2.5%)	3 (2.2%)	1 (5.0%)	2.4	0.2–23.8	0.469
Laboratory test results						
CRP (mg/dL)	3.9 (2.3–8.6)	3.9 (0.1–29.4)	3.9 (1.2–78.2)	1.0	1.0–1.1	0.175
Leukocyte (×109/L)	8.2 (5.2–11.9)	8.2 (4.7–12.1)	8.3 (6.2–11.1)	1.0	0.9–1.1	0.706
Neutrophils (×109/L)	6.5 (3.6–9.5)	6.6 (3.3–9.9)	6.3 (4.1–9.1)	1.0	0.9–1.1	0.643
ALT (U/L)	24.6 (13.5–41.9)	24.4 (3.7–147.0)	30.2 (1.7–419.1)	1.0	1.0–1.0	0.024*
SCr (μmol/L)	63.5 (51.3–83.6)	62.1 (22.4–1392.0)	69.0 (37.9–791.0)	1.0	1.0–1.0	0.690
Appropriate empirical antibiotic therapy	132 (84.1%)	117 (85.4%)	15 (75.0%)	0.5	0.2–1.6	0.242
Vancomycin	40 (25.5%)	39 (28.5%)	1 (5.0%)	0.1	0.0–1.0	0.052
Carbapenems	33 (21.0%)	27 (19.7%)	6 (30.0%)	1.7	0.6–5.0	0.296
Linezolid	4 (2.5%)	4	0	–	–	–

*OR* odds ratio, *95% CI* confidence interval, *CVC* central venous catheter, *MV* mechanical ventilation, *CRP* c-reactive protein, *ALT* alanine aminotransferase, *SCr* serum creatinine, *APACHE II* acute physiology and chronic health evaluation (ii), *qSOFA* quick sepsis related organ failure assessment

*Bold font means that the risk factor was statistically *p* < 0.05 in the univariate logistics analysis and was included in the multivariate logistics analysis

At the onset of CoNS bacteraemia, 42.0% (66/157) of patients were residents in the ICU and the most common comorbidities were cardiovascular disease (60.5%, 95/157).

Prior to the onset of CoNS bacteraemia, the median length of total hospital stay was 15.0 days (IQR, 4.0–35.0 days), and the length ≥ 28 days accounted for 33.8% (53/157). In addition, the most common treatments were indwelling CVC (56.1% 88/157) within 7 days prior to the onset of CoNS bacteraemia.

Within 24 h after the onset of CoNS bacteraemia, the levels of ALT and SCr and the inflammatory markers including the number of leukocytes, number of neutrophils and the level of CRP exhibited in Table 2. Additionally, evaluated within this 24 h period, the APACHE II score and the qSOFA scores used to assess the severity of patients were also exhibited in Table 2.

Within 72 h after the onset of CoNS bacteraemia, empirical antibiotic therapy was appropriate among 132 patients (84.1%, 132/157). VAN, which was used for empirical antibiotic therapy, accounted for 25.5% (40/157), carbapenems accounted for 25.5% (40/157), and LIN was only used among 4 patients.

### Outcomes and risk factors for poor outcome

Most of our data regarding outcome were collected by reviewing the medical database, but there were 29 patients whose 30-day survival data were not available, because their hospital stay length did not reach 30 days from the onset of CoNS bacteraemia to discharge and we had followed up these 29 patients (18.5%, 29/157) to collect the 30-day survival data by telephone. Our data showed that the 30-day mortality of the 157 patients with CoNS bacteraemia was 12.7% (20/157), and the median survival length from the onset of CoNS bacteraemia was 17.0 days (IQR, 7.8–21.2 days) among the 20 non-survival patients.

Univariate logistics analysis of risk factors associated with 30-day mortality of CoNS bacteraemia were also shown in Table 2. The results suggested that the 30-day mortality was possibly associated with age ≥ 60 years (OR 3.2), residence in the ICU (OR 3.2), qSOFA scores (2 and 3) (OR 3.2), APACHE II score (OR 3.2), ALT (OR 3.2) and total hospital stay length prior to the onset of CoNS bacteraemia (≥28 days) (OR 3.2). In addition, the comorbidities of pneumonia (OR 3.2), chronic renal failure (OR 3.2) and chronic liver failure (OR 3.2) were also associated risk factors. All the possible risk factors above were re-analysed in multivariate logistics analysis to find the independent risk factors. Additionally, although appropriate empirical antibiotic therapy was not a statistically significant variable (OR 0.5, 95% CI 0.2–1.6, *p* = 0.242) in our study, it was still included in the multivariate analysis since appropriate empirical antibiotic therapy was a clearly protective factor for outcome of infectious diseases in many previous studies. For example, appropriate antimicrobial therapy was the significant protective factor (OR 0.33, *p* = 0.013) in enterococcal bloodstream infections [19].

Before these variables above were included in the multivariate regression analysis, we performed collinearity and correlation analysis between these variables to exclude the composite variables. The multicollinearity results showed that the Tolerance of each variable was> 0.2 and the Variance Inflation Factor (VIF) of each variable was< 10 and the largest VIF was 2.150 (APACHE II score), indicating that there was no multicollinearity between these risk factors. However, exhibited in Table 3, the correlation analysis showed that the spearman correlation coefficient (r) between the APACHE II score and the qSOFA score was big (r = 0.594, *p* < 0.05). In addition, shown in Table 3, the APACHE II score was also significantly correlated with other factors, such as age (r = 0.281), residence in ICU (r = 0.333), chronic renal failure (r = 0.282) and so on. Additionally, there are some same parameters used to calculate the value of the APACHE II score and the qSOFA score, and the qSOFA score is part of the APACHE II score. Hence, we believed that the APACHE II score might have multicollinearity with other factors and affect the statistical results. Therefore, we excluded the APACHE II score from the multivariate regression analysis. Similarly, as shown in Table 3, there was a significant correlation between ALT and chronic liver failure, although the correlation coefficient was small (r = 0.339, *p* < 0.05). Additionally, taking into account the close relationship between chronic liver failure and the level of ALT in clinical practice, we thought they might be composite factors and we also ruled out the ALT from the multivariate regression analysis.

**Table 3:** The correlation coefficient between factors planed to be included in the multivariate regression analysis model

Correlation coefficient (r)	APACHE II score	ALT	Age ≥ 60 years	Residence in ICU	qSOFA (2 and 3)	Prior hospital stay length (≥28 days)	Pneumonia	Chronic liver failure	Chronic renal failure
Appropriate empirical antibiotic therapy	−0.030	0.131	0.094	−0.017	0.072	−0.057	−0.153	0.028	−0.032
Chronic renal failure	0.261*	−0.007	0.006	0.125	0.073	−0.111	0.049	0.218*	1.000
Chronic liver failure	0.202*	0.339*	0.000	0.127	0.131	0.007	0.116	1.000	/
Pneumonia	0.330*	0.055	0.265*	0.267*	0.251*	0.280*	1.000	/	/
Prior hospital stay length (≥28 days)	0.262*	−0.109	0.217*	0.238*	0.251*	1.000	/	/	/
qSOFA score (2 and 3)	0.594*	0.033	0.120	0.334*	1.000	/	/	/	/
Residence in ICU	0.333*	0.067	0.061	1.000	/	/	/	/	/
Age (≥60 years)	0.281*	−0.107	1.000	/	/	/	/	/	/
ALT (U/L)	0.033	1.000	/	/	/	/	/	/	/

*ALT* alanine aminotransferase

*Bold font means that correlation coefficient (r) was statistically *p* < 0.05 and there was a significant correlation between the two variables

Finally, only 8 risk factors were included in the multivariate regression analysis (shown in Table 4). About sample size estimation in regression analysis, we had used the previous empirical method to assess whether our sample size was sufficient. In regression analysis, the sample size used for prognostic risk factor analysis requires at least 10 individuals for each prognostic factor. Because we included 8 risk factors in the multivariate regression analysis in our study (Table 4), the sample size required at least 80 patients. In our study, we included 157 patients and the sample size should theoretically be able to meet statistical needs. Because we did not perform power analysis to estimate the sample size before the statistical analysis, we could not report a post hoc power calculation.

**Table 4:** The multivariate regression analysis of risk factors for 30-day mortality of patients diagnosed as CoNS bacteraemia

	Logistics regression^a^			Cox regression		
	OR	95% CI	*p*-value	HR	95% CI	*p*-value
Age						
≥ 60 years	3.3	0.9–12.6	0.084	2.6	0.8–8.4	0.100
< 60 years						
Residence in ICU	1.7	0.5–5.6	0.399	1.6	0.6–4.8	0.371
qSOFA score						
2 and 3	2.8	0.9–9.4	0.089	2.1	0.8–5.6	0.143
0 and 1						
Prior hospital stay length						
≥ 28 days	2.6	0.8–9.0	0.132	1.9	0.7–5.2	0.184
< 28 days						
Pneumonia	1.2	0.4–4.1	0.756	1.2	0.4–3.5	0.687
Chronic liver failure	4.0	1.2–13.1	0.024*	2.9	1.1–7.6	0.026*
Chronic renal failure	5.9	1.6–21.5	0.007*	3.9	1.5–10.5	0.007*
Appropriate empirical antibiotic therapy	0.3	0.1–1.1	0.066	0.3	0.1–1.0	0.060

^a^The predicted values of the final model was 91.7%. *OR* odds ratio, *HR* hazard ratio, *CI* confidence interval

*Bold font means that the risk factor was statistically *p* < 0.05 and was the independent risk factor among the 8 factors

At first, we had performed multivariate logistic regression analysis and the predicted values of the regression model was 91.7%. The results suggested that chronic renal failure (OR 5.9, 95% CI 1.6–21.5, *p* = 0.007) and chronic liver failure (OR 4.0, 95% CI 1.2–13.1, *p* = 0.024) were both the significant independent risk factors for the 30-day mortality of CoNS bacteraemia in the regression model (Table 4).

Then, taking into account the influence of the time to event, we also performed multivariate Cox regression analysis. The Cox regression analysis showed that chronic renal failure (HR 3.9, 95% CI 1.2–13.1, *p* = 0.007) and chronic liver failure (HR 2.9, 95% CI 1.1–7.6, *p* = 0.026) were still independent risk factor for the 30-day mortality of CoNS bacteraemia in the regression model (Table 4).

## Discussion

CoNS bacteraemia is difficult to diagnose because the majority of CoNS-positive blood cultures are usually considered contamination or non-pathogens. The true rate of CoNS bacteraemia ranged from 5 to 39.6% [13, 20, 21]. In this study, a total of 1241 adult patients with CoNS-positive blood cultures were identified. However, the majority were excluded as contamination, only 157 (12.7%) patients were finally confirmed as having CoNS bacteraemia, and their first CoNS-positive blood cultures were included in the study.

Among the 157 patients, the top two common species of CoNS were *Staphylococcus hominis* (40.8%) and *Staphylococcus epidermidis* (36.3%). This result was consistent with previous studies, although the proportion of *Staphylococcus epidermidis* demonstrated as the most common species of CoNS [4, 22]. Additionally, we found several rare species of CoNS, including three isolates of *Staphylococcus saprophyticus*, two isolates of *Staphylococcus cohnii* and 1 isolate of *Staphylococcus sciuri*.

Regarding CoNS resistance, CoNS bacteraemia species showed a multi-drug resistance profile in many previous studies [11, 23, 24]. In our study, 12 antibiotics were tested for antimicrobial susceptibility. As shown in Table 1, among the 157 patients, resistance to penicillin (94.9%), oxacillin (93.6%) and erythromycin (92.4%) was found in almost all species of CoNS, and the three antibiotics could not be recommended for empirical antibiotic therapy of CoNS bacteraemia. The resistance to cefoxitin (78.3%), clindamycin (73.2%), levofloxacin (72.6%) and trimethoprim/sulfamethoxazole (59.9%) were slightly lower but still very high, and these antibiotics must be cautiously selected for anti-CoNS bacteraemia. OXA was used as the indication for methicillin resistance; thus, 93.6% of the 157 species of CoNS were considered methicillin resistant, and 96.5% of *Staphylococcus epidermidis* were able to be defined as MRSE. This result was similar to research data in southwestern Saudi Arabia [13] and other countries [23, 25]. Not surprisingly, consistent with most previous studies, resistance to vancomycin or linezolid was not found [4, 26]. Multi-drug resistance has made vancomycin the first choice for anti-CoNS, and 25.5% (40/157) of patients were prescribed VAN for empirical antibiotic therapy within 72 h after the onset of CoNS bacteraemia in this study (Table 2). However, VAN-resistant CoNS does exist, for example, Anne Santerre Henriksen et al. reported that 1 out of 460 isolates displayed resistance to VAN in their study [27]. For this reason, although VAN-resistant CoNS was not present in our study, we still inferred that VAN-resistant CoNS bacteraemia may appear, and it deserves our attention in the clinic. In addition to the above, we were surprised to find that CoNS resistance to gentamicin (22.3%) and rifampicin (10.8%) was relatively low, and these antibiotics could be considered as alternative drugs for clinical anti-CoNS treatment in some proper patients, in addition to vancomycin and linezolid.

In addition to species distribution and antimicrobial resistance, we also analysed the risk factors for poor outcome among the 157 patients in this study. As shown in Table 2, our results suggested that patients aged ≥60 years (58.6%) and males (68.8%) accounted for the majority. The 30-day mortality of patients with CoNS bacteraemia was up to 12.7% (20/157). We observed in univariate analysis that the 30-day mortality was possibly associated with 9 possible risk factors such age ≥ 60 years (Table 2). After ruled out the possible composite factors by the correlation and multicollinearity analysis (Table 3), we selected 8 factors to be included in the multivariate regression analysis (Table 4). The multivariate logistics regression analysis suggested that chronic renal failure and chronic liver failure were statistically significant independent risk factors, and the results were verified by multivariate Cox regression analysis (Table 4). Thus, the results indicated that the 30-day mortality of patients with chronic renal failure was 5.9 times than that of patients without chronic renal failure (OR 5.9, 95% CI 1.6–21.5, *p* = 0.007) after the onset of CoNS bacteraemia. Similarly, the 30-day mortality of patients with chronic liver failure was 4.0 times than that of patients without chronic liver failure (OR 4.0, 95% CI 1.2–13.1, *p* = 0.024).

The results were understandable and acceptable, since a large number of studies on chronic renal failure had suggested that infection was a clear risk factor for acute exacerbation and death in chronic renal failure. For example, the high rate of infectious complication was found to be a major determinant of 2-year mortality after coronary artery bypass grafting in patients with chronic renal disease (HR 4.42–9.39) [28]. Conversely, chronic renal failure was also a risk factor for 30-day mortality in patients with catheter-related bloodstream infection (OR 11.1) [29] and in patients with *Pseudomonas aeruginosa* bacteremia (OR 4.93) [30].

Similarly, infections was also one of the most common precipitating factors in acute-on-chronic liver failure [31]. For example, Junjun Cai et al. found Infections (regardless of first or second infection) can increase the 90-day mortality significantly in patients with acute-on-chronic liver failure and the presence of second infection were independent risk factors (OR 2.37) for 90-day mortality in acute-on-chronic liver failure after the first infection [32]. On the other hand, chronic liver failure was also a risk factor for death in bacteraemia. For example, chronic liver failure was an independent risk factors (OR 3.3) associated with 30-day mortality in enterococcal bacteraemia [19].

Our results had also exhibited similar conclusions in our study. We believed that the onset of CoNS bacteraemia could promote the aggravation of chronic renal failure (or chronic liver failure), and conversely, the risk of 30-day mortality of patients with chronic renal failure (or chronic liver failure) was inevitably higher than that of patients without chronic renal failure (or chronic liver failure) after the onset of CoNS bacteraemia. From these perspectives, our data results were just the reflection of these clinical phenomena rather than accidental results.

In addition to chronic renal failure and chronic liver failure, our results also showed that age ≥ 60 years (OR 3.3, *p* = 0.084), residence in ICU (OR 1.7, *p* = 0.399), qSOFA (2 and 3) (OR 2.8, *p* = 0.089) and pior hospital stay length ≥ 28 days (OR 2.6, *p* = 0.132) were also the risk factors for 30-day mortality, and appropriate empirical antibiotic therapy was the protective factor (OR 0.3, *p* = 0.066). They were statistically significant independent risk factors or protective factors in some previous studies of infectious diseases although these factors were not significant in our study. The reason for the lack of significance of these factors might be that the sample size was still not sufficient in our study.

In conclusion, we analysed the species distribution, antibiotic resistance and risk factors for 30-day mortality in 157 patients with CoNS bacteraemia. We found that most species were *Staphylococcus hominis* and *Staphylococcus epidermidis*. All CoNS had high antibiotic resistance, 93.6% were resistant to methicillin, and none was resistant to vancomycin or linezolid. Additionally, our results had also demonstrated that chronic renal failure and chronic liver failure were the independent risk factors for 30-day mortality of CoNS bacteraemia. Patients with chronic renal failure or chronic liver failure have a higher 30-day mortality after the onset of CoNS bacteraemia and their treatments should be attracted more attention to prevent and reduce the occurrence of death as much as possible.

### Limitations

Several limitations also existed in the study. First, this study was retrospective and observational, and some unmeasured confounding factors might exist. For example, antimicrobial susceptibility to carbapenems was not tested according to the 2014 CLSI standards, but 32 out of 157 patients (21%) were given carbapenems for empirical antibiotic therapy, and their effectiveness could be assessed only through clinical symptoms rather than drug susceptibility testing. This might have produced small errors, but we consider it to have little effect on the analysis of risk factors for mortality. Second, the number of patients was limited in this study in a single centre. Although 1241 patients had CoNS-positive blood cultures, only 157 patients were included in the study, and other CoNS-positive blood cultures were mainly considered as being contaminated. However, we found that some mild patients with true CoNS bacteraemia might recover quickly after the first CoNS-positive blood culture, but no additional blood culture was drawn. Therefore, some mild patients with true CoNS bacteraemia may be mistakenly excluded from the study. This may lead to overestimation of the severity and the 30-day mortality of CoNS bacteraemia. However, this is clinically acceptable because it helps reduce medical expenses and overuse of antibiotics by reducing excessive attention to patients who are mild and recover quickly.

## Abbreviations

95% CI: 95% confidence interval
ALT: Alanine aminotransferase
APACHE II: Acute physiology and chronic health evaluation (ii)
CEF: Cefoxitin
CLN: Clindamycin
CLSI: Clinical and Laboratory Standards Institute
CoNS: Coagulase-negative staphylococci
CRP: C-reactive protein
CVC: Central venous catheter
ERY: Erythromycin
GEN: Gentamicin
IQR: Interquartile range
LEV: Levofloxacin
LIN: Linezolid
MOX: Moxifloxacin
MRSE: Methicillin-resistant *Staphylococcus epidermidis*
OR: Odds ratio
OXA: Oxacillin
PEN: Penicillin
PLAGH: Chinese People’s Liberation Army General Hospital
qSOFA: Quick sepsis related organ failure assessment
RIF: Rifampicin
SCr: Serum creatinine
SUL: Trimethoprim/sulfamethoxazole
VAN: Vancomycin
